# Medical Opinion and Movement

**Published:** 1908-04-18

**Authors:** 


### Medical Opinion and Movement.

Eclampsia is so complex a condition, and the treatment involves likewise so many considerations that it is difficult to ascribe recovery in a case to any special measure of the many to which one naturally resorts. Lumbar puncture appears, however, to have justified itself on physiological reasoning as well as by the favourable results following its adoption. It was originally proposed by Helme. The high arterial tension almost invariably present in the eclamptic state suggested also a hypertension of the cerebro-spinal fluid as the cause of the convulsions, loss of vision, and other cerebral symptoms. Kronig has shown that this hypertension may amount to 200 or even 500 millimetres pressure in conditions of eclampsia. It is therefore only reasonable to suppose that a reduction of this pressure would cause an amelioration of the cerebro-spinal symptoms. It must, however, be borne in mind that lumbar puncture in these conditions is only palliative and cannot attack the cause, which is essentially of a toxæmic nature. It must therefore be used in conjunction with other recognised measures. The convulsions of eclampsia are often the most urgent symptoms, and as a weapon against these lumbar puncture probably offers the most immediate relief.

Colloid preparations of the metals are receiving considerable attention as therapeutic agents by several French authorities. Their efficacy depends upon the peculiar physical properties of a colloid solution. Their physical and physiological properties offer a wide field for research, in which several French investigators, notably Victor Henri, Charrin, and Robin, have already made notable progress. Physically a colloid solution consists of a substance in suspension in ultra-microscopic parties, and it is the infinitesimal size of the particles upon which depend the peculiar active properties of these solutions. They are electrically charged, and they possess a chemical activity comparable to that of the ferments and toxines. They are catalytic, and their power of catalysis can be measured by the proportion of hydrogen peroxide decomposed in a given time and at a certain temperature. This catalytic power varies with different colloids and also for the same colloid, according to the size of the particles. The more infinitesimal the particles the greater the catalytic power. A further property of these colloids to be mentioned is their affinity for one another and power of combination and interaction. It is now recognised that these peculiar properties of colloid solutions, their chemical activity, their catalytic power, and their capacity to combine, form the basis of organic cell activity—of metabolism; of the action of antitoxins on toxins; of the phenomena of agglutinins; and of the digestion of albumin by the ferments. From these conclusions the suggestion naturally follows that the reactions of the organism may be most easily modified by such colloid preparations.

The colloid preparations which have been specially exploited for therapeutic purposes are those of the metals—silver, gold, platinum and palladium—and of these the most active is silver. The finest and, therefore, the most active solution is obtained by the electric current. Experiments *in vitro* with the microbes of disease have shown that an infinitesimal amount of such a preparation is sufficient to hinder the development of, or completely sterilise, a bacterial culture. The anthrax bacillus, for instance, cannot grow in a medium containing 1/50,000 of colloid silver. Experiments on white mice have shown that injections of colloid silver solutions can prevent a fatal result from lethal doses of the pneumococcus. Following these experiments the colloid preparations have been employed therapeutically both for local lesions and for general infections with encouraging results. MM. Chirié and David have reported several cases of mammary abscess treated successfully by aspiration and by local injections of colloid silver solution, and they also claim to have cured two cases of puerperal fever by intravenous injections. Gaillard has found a markedly favourable effect in typhoid fever by daily injections. After a series of four to eight injections there is a rapid fall of temperature, accompanied by abundant diuresis. Favourable results have also been reported in pneumonia, rheumatic fever, and influenza, but the preparations of silver and gold do not appear to have any effect in pulmonary tuberculosis. These colloid preparations of the metals afford a subject of considerable interest. Their employment is based upon the most advanced knowledge of the physical and physiological properties of matter, and they appear to offer a valuable addition to our medicinal armamentarium.

Professor Rubner, of Berlin, has made a special study of problems relating to development and growth, and some of his deductions and generalisations are highly interesting. He maintains that the main factor influencing the growth of the child is the inherited rate of cell proliferation and cell division. The quantity of calories provided by the food ingested is only of secondary importance. The weight of the newly-born creature is doubled by growth in very different intervals of time in different animals. The newly-born kitten doubles its weight in nine days, the calf in forty-seven days, while the human infant requires 180 days. The quantity of energy necessary to bring about this doubling is remarkably constant for the animals, being about 4,000 calories per kilogramme of the body-weight, but in man it amounts to 29,000 calories. The slow growth of the human body is perhaps compensated by the greater development of brain. Man has moreover an exceptional position in regard to the duration of life and its relation to the length of the period of growth. Whilst the horse lives thirty-five years, the cow thirty years, and the dog fifteen years, periods of growth are respectively five, four, and two years—that is, from one-sixth to one-seventh of the total duration of life. In man a total duration of life of seventy to eighty years is associated with a period of growth of twenty to twenty-four years—that is, one-third to one-fourth. It has recently been suggested that under favourable conditions man's full span of life should extend to 100 years or more, and this extension would certainly appear to be justified by a comparison with the relative figures of growth and life of the lower animals.

---

Bittner, in an interesting paper in the "Deutsche Med. Wochenschrift," gives a *résumé* of several cases of scoliosis treated by Klapp's method. The children were encouraged to continue the exercises, which consist chiefly in "marching like a dog" (*i.e.* on all fours), making "swimming motions" while lying on the back or on the stomach for a long time each day, such exercises being carefully graded to the strength of each patient. The results were most encouraging, and the author speaks highly of the treatment. In adult cases it has, however, been disappointing. The treatment is easy to carry out, and the children can usually be interested in the exercises and need little supervision. The method is applicable to all cases in which there are no signs of active mischief, but is contra-indicated when the patient is feverish or when there is, in addition to the lateral curvature, any signs of kyphosis.

---

The Report of the Chicago Pasteur Institute on the cases of hydrophobia treated therein during the seventeen years of its existence is instructive and encouraging. Up to the end of last year more than three thousand patients had received the anti-hydrophobic treatment, in consequence of bites received from suspected animals. Ninety per cent. of these animals were dogs; while thirty-eight patients were infected by human beings suffering from hydrophobia. More than one-half of the infecting animals were proved experimentally to be rabid, while the great majority of the remainder had suffered clinically from rabies. Only seven deaths are reported amongst 3,130 patients treated—a mortality of 0.22 per cent. This is a highly satisfactory record.

---

The hymen is considered anatomically, medico-legally, and historically in an interesting paper by Dr. E. G. McKee in the *American Journal of Obstetrics* for March. From the point of view of medical jurisprudence the hymen is still of undoubted importance, although the evidence to be obtained therefrom is seldom conclusive. What Dr. McKee describes as the "folding hymen" is an anomalous condition which may lead medical witnesses into serious errors of judgment unless its possibility is remembered. Here the membrane is normal in appearance and structure, but of peculiarly yielding nature, readily admitting the entrance of an ordinary vaginal dilator or a fair sized speculum. During the passage of these instruments the hymen folds back against the vaginal wall and returns quite intact on their removal. Another anomaly is the Hymen Denticulatus, a fairly frequent condition, distinguished by a number of tooth-shaped indentations limited to the smooth inner border of the membrane, and especially found on its anterior surface. In this case the medical man may think that the hymen has been ruptured, unless he notes carefully that the borders are uniformly soft, and that there is no cicatrisation. According to the author, the principal varieties of hymen, arranged in ascending order of resistance to rupture, are as follows: Cribriform, semilunar, horseshoe, annular, bilobate, and imperforate. Dr. McKee quotes many curious cases, showing on the one hand the ease with which many hymens give way under the slightest accidental violence, and on the other the possibility of frequent coition and even delivery taking place with unruptured hymen.

---

At a meeting of the Therapeutical and Pharmacological Section of the Royal Society of Medicine, on March 24, Dr. John Milne Bramwell delivered an address on Hypnotism. He described the gradual evolution of hypnotic theory from the time of Elliotson and the later mesmerists to the present day. Mesmerism, Dr. Bramwell pointed out, was the first anæsthetic, and hypnotic anæsthesia is still occasionally induced at the present day. At the same time he regards this as being more of scientific than practical interest, for only a limited number of persons can be hypnotised deeply enough for operative purposes. Dr. Bramwell gave an account of his own work and drew attention to the fact that formerly his proportion of successes had been greater, when he was able to choose his cases from amongst the patients of his own general practice. At present, when the cases sent to him are almost invariably long-standing ones, in which other methods of treatment have been tried without success, his difficulties are considerably increased. Still, even in such cases success is often striking, and sometimes rapidly obtained. Dr. Bramwell rarely finds it necessary to induce deep hypnosis: a restful, drowsy state seems to be most favourable to the action of suggestion.

---

Tcherkesson draws attention to the utility of injections of sulphate of atropine in cases of strangulated hernia. The author uses small doses (two milligrams) injected subcutaneously. The patient is placed on a hard table and an ice compress is applied over the hernia, and half an hour after the injection has been given an attempt is made to reduce the hernia by gentle taxis. It is often advantageous to place the patient in the genu-pectoral position, and to give a second injection if there is further difficulty, but the attempts at reduction should be made with the utmost gentleness. The method is indicated as a preliminary to operation in cases which are seen very early, and also in those cases in which the patients or their friends refuse operative interference, or where, for some reason, such interference is contra-indicated. If the attempt is unsuccessful cœliotomy can always be resorted to, since the injections do not affect the necessary anæsthesia. The author, however, strongly urges that every strangulated hernia should be operated upon, even in cases where this method has been successful.

AN interesting old document, the record of an action for the payment of fees due to a medical practitioner in the year 1393, has just been published by Prof. Baas, of Karlsruhe. The action was tried, as it appears, before the Episcopal Court at Constance, and was brought by one "Konradus Bolling, physicus Constansiensis et capellanus altaris Sanctæ Fidis in ecclesia Constansiensi" against Johann Burgouver. The applicant's plea set forth that he had "diligenter, fideliter, utiliterque" treated the defendant," who had received an internal injury—"intus corpore confractus exstitit." Defendant, it is interesting to note, did not carry out the rules of treatment, "whereby," as plaintiff avers, "the cure was considerably delayed." The treatment is carefully described, and seems to have consisted in "signis ac procnosticationibus attentis," right median phlebotomy, and compresses of olive oil over the site of the abdominal (?) lesion. Plaintiff had also given a recipe or prescription for an "analgesic balsam," and "divina gratia auxiliante" had succeeded in curing the defendant. The defendant, in his replication, denied liability for the honorarium, declaring that the plaintiff, as a priest, was not a qualified medical man, and that he had not treated defendant properly. No counterclaim for *mala praxis* was put in, but the defendant energetically stated that if plaintiff had been a layman, and not a frocked priest, the proper honorarium for him would have consisted "in a severe and general flagellation." Unfortunately the result of this interesting action has not come down to us. The documents are incomplete, and the proceedings seem to have been interrupted at the most interesting stage. Prof. Baas concludes that the Court found itself on the horns of a dilemma, and probably dismissed the case.

PROFESSOR HEILBRONNER, of Utrecht, in a recent article in the *Münchener Medizinsche Wochenschrift*, deals at length with the question of the legal liability of the drunkard. The subject is an interesting one, and has already formed the basis of a large number of articles, among which may be mentioned the monograph from the pen of Prof. Hoppe on "Alcohol and Criminality," which was published in 1906. Prof. Heilbronner discusses the various positions which authorities have taken up, some in favour of and others again very much against the alcoholic, and comes to the conclusion that, as far as the medical practitioner is concerned, there are two questions which demand special attention. These are: Is it the duty of the medical man to claim that, in each case where an action of a drunkard is involved, expert opinion should be demanded as to the liability of the accused; and, secondly, if such expert evidence is not necessary, or, at all events, not indispensable, is it his duty to guide the court, or to help it in coming to a conclusion as to how far the accused is a criminal? These two questions are intimately connected, and their discussion opens up a wide field for controversy and disputation. Equally important is the question of "proportionate liability," which the author also attempts to discuss. Psychiatry has only recently succeeded in winning respect and recognition from the lawyers, and in some subjects it still has a hard struggle before it to obtain recognition for certain well-established propositions of which the law does not at present take cognisance. At the same time, as the author justly remarks, psychiatrists and medical psychologists should take care not to dogmatise on matters which are still in doubt. One of such matters is the legal responsibility of the drunkard or the alcoholic, in discussing which the practical points at issue ought never to be lost sight of in the mass of purely scientific interests.

CASES of so-called "acute idiopathic phlegmonous gastritis" are exceedingly rare, and lately a good deal of doubt has been expressed as to whether such cases are really primary inflammations of the stomach. The latest word on the subject emanates from Hosch, of the Pathological Institute at Basle, who has collected some eighty-three published cases of this affection. A large number of the published cases, as he shows, were clearly cases of secondary phlegmonous gastritis, due to ascending (duodenal ulcer) or descending (acute œsophagitis) causes. A number of cases, however, remain, in which it is exceedingly difficult to trace the source of infection. Post-mortem, such cases show extensive phlegmonous inflammation of the gastric tissue, involving not only the mucosa and submucosa, but the muscular and serous layers as well. These cases cannot be considered as local gastric abscesses, as some authors suggest that they should be regarded, for in most cases the inflammatory lesions are not circumscribed, but very diffuse, involving the whole stomach, from the cardiac end down to the pylorus.

CLINICALLY, such cases are of great importance, and their diagnosis is often a matter of great difficulty. The author, for instance, reports an almost typical case, so far as post-mortem findings went, in which prominent gastric symptoms were not observed. The course and symptoms of the case suggested a peritonitis; the patient had no hiccough and vomiting was by no means a prominent symptom. The abdomen was opened, and it was found that the peritoneal cavity contained some purulent fluid. No perforation or special lesion in any viscus could be discovered. The patient never rallied from the operation, and died soon afterwards. At the autopsy a condition of phlegmonous inflammation of the stomach was found. Cases of true gastric abscess (that is, an abscess located in the stomach wall) are very rare, and the author thinks that the probability of the lesion being an abscess and not a phlegmonous gastritis is only to be considered when the patient has definitely brought up quantities of pus in the vomit. The therapeutics of the lesion the author does not discuss—they can hardly be said to exist—but he warns strongly against lavage of the stomach in all cases in which a phlegmonous inflammation is considered as one of the probabilities.

